# Supplementary material for: Relationship between Recovery from COVID-19-Induced Smell Loss and General and Oral Health Factors
Source: Medicina (Kaunas). 2022 Feb 14;58(2):283. doi: 10.3390/medicina58020283 (PMC8877343; doi:10.3390/medicina58020283)
Supplement: Supplementary file 1 [file medicina-58-00283-s001.zip › medicina-1603454-supplementary.pdf]

medicina

Supplementary Material

Relationship between Recovery of COVID-19 Induced Smell and Taste Loss and General and Oral Health Factors

Georgia Catton and Alexander Gardner *

Department of Restorative Dentistry, Dundee Dental Hospital and School, University of Dundee, Dundee DD1 4HR, UK; g.s.catton@dundee.ac.uk
* Correspondence: agardner001@dundee.ac.uk

Supplementary Data S1: Information for participants and questionnaire.

Study Description: We are conducting a survey of the loss of taste and smell in people who have experienced COVID-19 infection. This is a short survey that takes about five minutes to complete. It is open to anyone over the age of 18 who has been diagnosed with COVID-19 or suspected symptomatic COVID-19 and who can read and write in English. **Even if you did not experience any loss of taste and smell your participation is extremely valuable.**

It is hoped that the resulting data can allow further insight into the significance of loss of taste and smell as symptoms of COVID-19 infection.

Study Link: https://dundee.onlinesurveys.ac.uk/covid-19-smell-and-taste-questionnaire

Disclaimer (ethical/legal): All responses are anonymous and entirely voluntary. We do not collect any identifiable data. Once a survey has been completed it is no longer possible to remove the data from the master list as no identifiers will be present. Data will be stored confidentially and securely in accordance with University of Dundee data security and GDPR regulations. Data will not be accessible to those out with the research team. Data may be shared with other academics for purposes of future research. In this event data will be shared securely with a data transfer agreement in place. This study has been approved by the University of Dundee Schools of Nursing & Health Sciences and Dentistry Research Ethics Committee, application number: UOD\SDEN\STAFF\2020\017.

End Date: Open

1. How was your diagnosis of COVID-19 made?

- Self-diagnosed
- Diagnosed by a doctor
- Diagnosed by a positive PCR test

2. At the time of illness, what was your age in years?

3. What is your sex?

- Male
- Female

4. What is your ethnicity?

5. How would you rate the severity of your illness from 0 to 10?

(0 = no symptoms, 10 = most severe illness imaginable)

6. What was the duration of your illness and recovery in days?

7. What was you experience of loss of smell, rated from 0 to 10?

(0 = no loss of smell, 10 = complete inability to smell anything)

7b. How many days did it take for your sense of smell to return to normal?

8. What was you experience of loss of taste, rated from 0 to 10?

(0 = no loss of taste, 10 = complete inability to taste anything)

8b. How many days did it take for your sense of taste to return to normal?

8c. Was any specific taste affected more than others? Please provide a rating for each taste:

(0 = no loss of taste, 10 = complete inability to taste this sensation)

- Salt
- Sweet
- Sour
- Bitter
- Umami (savoury flavour)

9. What was you experience of nasal congestion (blocked nose) rated from 0 to 10?

(0 = no congestion, 10 = complete inability to breathe through nose)

10. Prior to your illness with COVID-19, how frequently would you typically experience a cough/cold/flu-like illness?

- Less than once a year

-about once a year

-two or three times a year

-four or five times a year

-more than five times a year

11. Prior to your illness, were you a smoker? If so, how much would you typically smoke?

12. Prior to your illness, did you drink alcohol? If so, how much would you typically consume?

13. Prior to your illness, what was your height? (please specify feet/inches or centimetres)

14. Prior to your illness, what was your weight? (please specify pounds or kilograms)

15. Prior to your illness, how many portions of fruit and vegetables would you eat per day?

16. Prior to your illness, what medication, including any vitamin or mineral supplements, were you taking? Please complete the table:

**Medication name**	**Dose per day**	**Length of time on medication**

17. Prior to your illness, how would you care for your teeth?

- How often would you brush your teeth?

- What toothpaste would you use?

- How often would you floss?

18. Prior to your illness, had you lost any teeth? If so, how many, and why were they lost?

19. Prior to your illness, how many fillings did you have?

- no fillings

- one to five fillings

- five to ten fillings

-more than ten fillings

20. Prior to your illness, did you wear any oral appliances?

- none

- upper retainer

-lower retainer

-upper and lower retainer

- upper denture

-lower denture

-upper and lower denture

Supplementary Table S1: Comparison of short (≤28 days) and long (>28 days) smell and taste loss only on participants with confirmed PCR test (n=118). For Fisher's exact test and Chi2 test observed values are shown followed by expected values in brackets.

**Comparison of short (≤28 days) and long (>28 days) smell loss**							
**Variable**	**Test**	**Distributions**				**Test-statistic**	**p-value**
		**Short (Mean ± 95% CI)**		**Long (Mean ± 95% CI)**			
Age	t-test	29.36 (27.55 – 31.17)		34.25 (30.12 – 38.38)		-2.49	0.014
Illness severity	Mann-Whitney	3.93 (3.51 – 4.35)		5.34 (4.69 – 5.99)		-3.37	0.001
Flossing	Fisher's exact	No	15 (19.2)	No	13 (8.8)	NA	0.042
		Yes	64 (59.8)	Yes	23 (27.2)		
